# Analysis of the prognostic utility of the cell cycle progression (CCP) score generated from needle biopsy in men treated with definitive therapy

**DOI:** 10.1038/s41391-019-0159-9

**Published:** 2019-06-27

**Authors:** Daniel J. Canter, Stephen Freedland, Saradha Rajamani, Maria Latsis, Margaret Variano, Shams Halat, Jonathan Tward, Todd Cohen, Steven Stone, Thorsten Schlomm, Jay Bishoff, Stephen Bardot

**Affiliations:** 1 Ochsner Clinic, Department of Urology, New Orleans, LA USA; 2 Queensland School of Medicine, Queensland, Australia; 3 Cedars-Sinai Medical Center, Los Angeles, CA and Durham VA Medical Center, Durham, NC USA; 4 Myriad Genetics, Inc., Salt Lake City, UT USA; 5 University of Utah Huntsman Cancer Hospital, Salt Lake City, UT USA; 6 Charité - Universitätsmedizin Berlin, Berlin, Germany; 7 Intermountain Urological Institute, Salt Lake City, UT USA

**Keywords:** Prostate cancer, Prognostic markers

## Abstract

**Background:**

Accurate risk stratification can help guide appropriate treatment decisions in men with localized prostate cancer. Here, we evaluated the independent ability of the molecular cell cycle progression (CCP) score and the combined cell-cycle clinical risk (CCR) score to predict 10-year risk of progression to metastatic disease in a large, pooled analysis of men with definitively treated prostate cancer.

**Methods:**

The pooled analysis included 1,062 patients from four institutions (Martini Clinic, Durham VA Medical Center, Intermountain Healthcare, Ochsner Clinic) treated definitively for localized prostate cancer by either radical prostatectomy or radiotherapy (brachytherapy or external beam radiotherapy ± hormone therapy). The CCP score was determined using the RNA expression of 46 genes from archival formalin-fixed paraffin-embedded biopsy tissue. The CCR score was calculated using a predefined linear combination of the CCP score and the Cancer of the Prostate Risk Assessment (CAPRA) score. The scores were evaluated for association with 10-year risk of metastatic disease following definitive therapy after adjusting for other clinical variables.

**Results:**

The CCP score was strongly associated with 10-year risk of metastatic disease in multivariable analysis [Hazard Ratio per unit score = 2.21; 95% confidence interval (CI) 1.64, 2.98; *p* = 1.9 × 10^−6^] after adjusting for CAPRA, treatment type, and cohort. CCR was also highly prognostic (Hazard Ratio per unit score = 4.00; 95% CI 2.95, 5.42; *p* = 6.3 × 10^−21^). There was no evidence of interaction between CCP or CCR and cohort (*p* = 0.79 and *p* = 0.86, respectively) or treatment type (*p* = 0.55 and *p* = 0.78, respectively). Observed patient CCR-based predicted risks for metastatic disease by 10 years ranged from 0.1 to 99.4%, (IQR 0.7%, 4.6%).

**Conclusions:**

Both CCP and CCR scores provided independent prognostic information for predicting progression to metastatic disease after both surgery and radiation. These results further demonstrate their potential use as a risk stratification tool in patients with newly-diagnosed prostate cancer.

## Introduction

The natural history of localized prostate cancer is highly variable, which can cause uncertainty in the selection of the appropriate management strategy for the individual patient [1, 2]. To help address this uncertainty, prognostic molecular biomarkers have emerged as important clinical adjuncts to standard clinicopathologic features to aid in evaluating the aggressiveness of newly diagnosed localized disease [3, 4]. To date, the primary clinical utility of biopsy-derived markers has been to improve identification of men with low-risk disease who may be good candidates for deferred treatment regimens like active surveillance [4–6]. However, biopsy-derived prognostic markers may also be useful in helping physicians personalize the intensity of therapeutic intervention for patients that need treatment. For example, the intensity of treatment could be altered or augmented if the patient’s expected risk of failing a specific treatment is predicted to be high based on pre-treatment risk stratification.

The Cell Cycle Progression (CCP) score is a well-validated prognostic RNA expression signature that is based on measuring the expression levels of 31 CCP and 15 housekeeping genes [7, 8]. The score improves risk discrimination compared to clinicopathologic features alone [7–12], and clinical utility studies have shown that physicians use the added prognostic information to help guide subsequent clinical management [13]. More recently, the molecular CCP score has been combined with the Cancer of the Prostate Risk Assessment (CAPRA) score into a validated prognostic model. This combined Clinical Cell-cycle Risk score (CCR score) provides a more precise estimate of risk than can be obtained using either variable alone [14].

Previous studies have focused on how the CCP and CCR scores can be used to help manage men who may be considering active surveillance [5]. Here, we evaluated the ability of these scores to predict clinical outcomes after definitive therapy. Specifically, we report on the association between both the CCP and CCR scores and 10-year risk of metastatic disease in a large pooled cohort of patients who underwent definitive therapy for localized prostate cancer.

## Methods

### Patients

Patients from the Martini Clinic (*N* = 162), Durham VA Medical Center (DVA; *N* = 131), Intermountain Healthcare (*N* = 123), and Ochsner Clinic (*N* = 646) were combined for this pooled analysis. The Martini Clinic, DVA, and Intermountain Healthcare cohorts have been previously described in detail [9]. In brief, the Martini Clinic cohort was randomly selected from a consecutive series of patients treated with radical prostatectomy (RP) at the Martini Clinic (Hamburg, Germany) from 2005 to 2006. Because the original diagnostic biopsies were unavailable, a simulated biopsy was generated by removing a tissue cylinder 0.6 mm in diameter from the region of the postoperative formalin fixed, paraffin embedded block containing the largest tumor foci. The DVA cohort included men who were treated with RP at DVA (Durham, NC) from 1994 to 2005. The Intermountain Healthcare cohort was treated with RP at Intermountain Healthcare (Salt Lake City, UT) between 1997 and 2004. The Ochsner Clinic cohort has also been previously described in detail and included a consecutive series of men treated at the Ochsner Clinic (New Orleans, LA) between 2006 and 2011 [15]. Institutional review board approval was obtained at all study sites. Men were included if they were treated for localized prostate cancer by either RP or radiotherapy [external beam radiotherapy (EBRT) ± androgen depravation therapy (ADT) or brachytherapy] and had complete molecular and clinicopathologic data.

### Molecular testing and CAPRA scores

All molecular data were generated blinded to patient outcomes. The CCP score was derived from the diagnostic biopsy or simulated biopsy (Martini Clinic only) at Myriad Genetics, Inc. (Salt Lake City, UT). CCP testing was performed as previously described [16, 17]. Briefly, formalin-fixed, paraffin-embedded needle cores with the largest extent of tumor were identified by an anatomic pathologist. The selected tissue regions were macrodissected and deparaffinized (Deparaffinization Solution, Qiagen, MD) and RNA extraction was performed using miRNeasy (Qiagen, MD). The gene expression for 31 CCP genes and 15 housekeeper genes was quantified in triplicate (TaqMan Low Density Arrays, ThermoFisher Scientific, MA).

The average expression of the CCP genes was normalized by the expression of the housekeeper genes to produce a CCP score [8]. The CCP score was combined with the CAPRA score (0.39 × CAPRA + 0.57 × CCP) to produce the CCR score [14]. CAPRA scores were derived from pre-surgical serum prostate-specific antigen (PSA) measurement, biopsy Gleason scores, clinical stage, percent positive needle cores, and age at diagnosis [18].

### Statistics

The primary endpoint in this analysis was progression to metastatic disease, which was confirmed by either a positive bone scan, whole body scan, computerized tomography, magnetic resonance imaging or plain X-ray. A previous combined analysis of the Martini Clinic, DVA, and Intermountain Healthcare cohorts by Bishoff et al. showed no evidence for an interaction between CCP score and cohort for predicting metastatic disease [9]. This included a specific analysis which excluded the Martini Clinic cohort (generated using simulated biopsy samples), which had no impact on the overall prognostic ability of the CCP score. As such, these three cohorts were considered as a single pooled cohort for this analysis (Bishoff cohort).

Descriptive statistics for continuous variables comparing the two cohorts were performed. Values expressed are the median and interquartile range (IQR; 25th and 75th percentiles). A multivariable Cox proportional hazards (PH) model was used to evaluate the prognostic value of the CCP score after accounting for other clinical covariates. CCP and CCR hazard ratios (HR with 95% CI) were per unit change in score. All *p*-values were two-sided. The CCR score-based risk curves were generated using Cox PH methods. Risk-cures were drawn at 7-years for individual cohorts due to limited events after that time point. Pooling the cohorts (to increase the number of late events) enabled evaluation of 10-year risk curves. The relative contributions of CCP and CAPRA for predicting metastatic disease in this cohort were compared to the pre-defined CCR model using a partial likelihood ratio test.

## Results

The final pooled cohort included 1,062 men: 416 men from the Bishoff cohort (Martini Clinic [*N* = 162], DVA [*N* = 131], and Intermountain Healthcare [*N* = 123]), and 646 men from the Ochsner Clinic cohort. All of the men were diagnosed with localized adenocarcinoma of the prostate and treated with either RP (*N* = 800) or radiotherapy (*N* = 262). The Ochsner and Bishoff cohorts were significantly different for all comparisons of clinical variables except pre-biopsy PSA (Table 1). However, the absolute differences were mostly minor. Median follow-up time for patients without events was 6.05 [Interquartile Range (IQR) 4.8, 8.0] years, and overall 3.3% (35/1,062) of patients progressed to metastatic disease (Table 1).

**Table 1:** Clinical characteristics according to cohort

	Ochsner Clinic (*N* = 646)		Bishoff Cohort (*N* = 416)		
Characteristic	*N*	Median (IQR) or frequency	*N*	Median (IQR) or frequency	*p*-value
Age at diagnosis (years)	646	64 (58, 70)	416	62 (58, 66)	2.0 × 10^−5^
**Ancestry**					
African-American	241	37.3%	67	16.1%	2.8 × 10^−14^
Non African-American	405	62.7%	349	83.9%	
Pre-biopsy PSA (ng/μL)	646	5.8 (4.5, 8.3)	416	6.0 (4.6, 9.0)	0.49
Biopsy Gleason Score^a^					
<7	333	51.5%	159	54.3%	3.2 × 10^−3^
3 + 4 = 7	156	24.1%	86	29.4%	
4 + 3 = 7	61	9.4%	28	9.6%	
>7	96	14.9%	28	6.8%	
Clinical T stage					
T1	471	72.9%	261	62.7%	1.4 × 10^−8^
T2	151	23.4%	154	37.0%	
T3	24	3.7%	1	0.2%	
Percent positive cores	646	42.9 (28.6, 66.7)	416	33.3 (20.0, 50.0)	1.3 × 10^−7^
CAPRA risk category					
Low (0–2)	288	44.6%	202	48.6%	3.2 × 10^−5^
Intermediate (3–5)	258	39.9%	187	45.0%	
High (6–10)	100	15.5%	27	6.5%	
CCP score	646	0.3 (−0.2, 0.9)	416	−0.1 (−0.6, 0.5)	1.5 × 10^−12^
Treatment					
Surgery	384	59.4%	416	100%	<2.2 × 10^−16^
XRT	262	40.6%	0	0%	
Progression to metastatic disease					
Events	28	4.3%	7	1.7%	<2.2 × 10^−16c^
Years to last follow-up^b^	646	5.5 (4.0, 6.8)	416	7.1 (5.4, 10.0)	
Events by AUA Risk Category^d^					
Low	2/285	0.7%	0/189	0.0%	n/a
Intermediate	9/200	4.5%	4/184	2.2%	
High	17/161	10.6%	3/43	7.0%	

*PSA* prostate-specific antigen, *CAPRA* cancer of the prostate risk assessment, *CCP* cell cycle progression, *XRT* external radiation therapy

^a^IHC cohort excluded from Bishoff cohort due to some patients missing secondary Gleason

^b^Follow-up time for men who had not experienced an event and were alive at the end of follow-up

^c^Wilcoxon rank sum *p*-value for follow-up time

^d^N shown as number of events over total number of patients within that risk category

On univariate analysis of the pooled cohort, the CCP score was strongly associated with progression to metastatic disease [Hazard Ratio (HR) per unit score = 2.93, *p* = 1.8 × 10^−11^], as were CAPRA score, treatment, and cohort (Table 2). However, only CCP score and CAPRA remained significant in a multivariable analysis that included all significant variables from univariate analysis (Table 2). There was no evidence for interaction between a patient’s CCP score and treatment (*p* = 0.55) or CCP score and cohort (*p* = 0.79). This indicates that the magnitude of the CCP HR for progression to metastasis was similar regardless of treatment type or cohort.

**Table 2:** Univariate and multivariable Cox models (*N* = 1062)

Variable	Hazard ratio^a^ (95% confidence interval)	*p*-value
Univariate analysis		
CCR score	4.00 (2.95, 5.42)	6.3 × 10^−21^
CCP score	2.93 (2.21, 3.90)	1.8 × 10^−11^
CAPRA	1.75 (1.53, 2.00)	4.2 × 10^−15^
Ancestry (AA/Non-AA)	0.62 (0.27, 1.43)	0.24
Treatment (Radiation/RP)	5.14 (2.58, 10.23)	4.5 × 10^−6^
Cohort	3.98 (1.64, 9.69)	6.1 × 10^−4^
Multivariable analysis for CCP^b^		
CCP score	2.21 (1.64, 2.98)	1.9 × 10^−6^
CAPRA	1.61 (1.37, 1.90)	1.3 × 10^−8^
Treatment (Radiation/RP)	1.36 (0.58, 3.20)	0.48
Cohort	1.63 (0.55, 4.78)	0.37
Multivariable analysis for CCR^b^		
CCR score	3.63 (2.60, 5.05)	2.1 × 10^−16^
Treatment (Radiation/RP)	1.33 (0.57, 3.11)	0.51
Cohort	1.64 (0.56, 4.83)	0.36

*CCR* cell-cycle clinical risk, *CCP* cell cycle progression, *CAPRA* cancer of the prostate risk assessment, *AA* African American, *RP* radical prostatectomy

^a^Hazard ratio per unit score for continuous variables

^b^Multivariable analysis performed separately for CCP and CCR scores because the CCR score is a linear combination of CCP and CAPRA

The CCR score was also highly prognostic for progression to metastatic disease for the combined cohort (HR per unit score = 4.0, *p* = 6.3 × 10^−21^; Table 2). The score remained highly significant after adjusting for treatment and cohort (Table 2). As observed for the CCP score, there was no evidence for an interaction between CCR and any other model variable (treatment *p* = 0.78; cohort *p* = 0.86). To further evaluate the impact of the cohort variable, we compared the HRs for CCR in each individual cohort (Supplemental Fig. 1). There was no evidence for HR heterogeneity in the CCR score. The CCR score was originally validated for the prediction of disease-specific mortality in conservatively-treated patients [14]. Nevertheless, the pre-defined model adequately accounted for all molecular and clinical information for predicting metastatic disease such that reweighting CCP or CAPRA did not add significant prognostic information to the CCR score (*p* = 0.69). The c-indices for progression to metastatic disease by 10-years were 0.790 for CCP, 0.857 for CAPRA, and 0.894 for CCR.

**Fig. 1:**
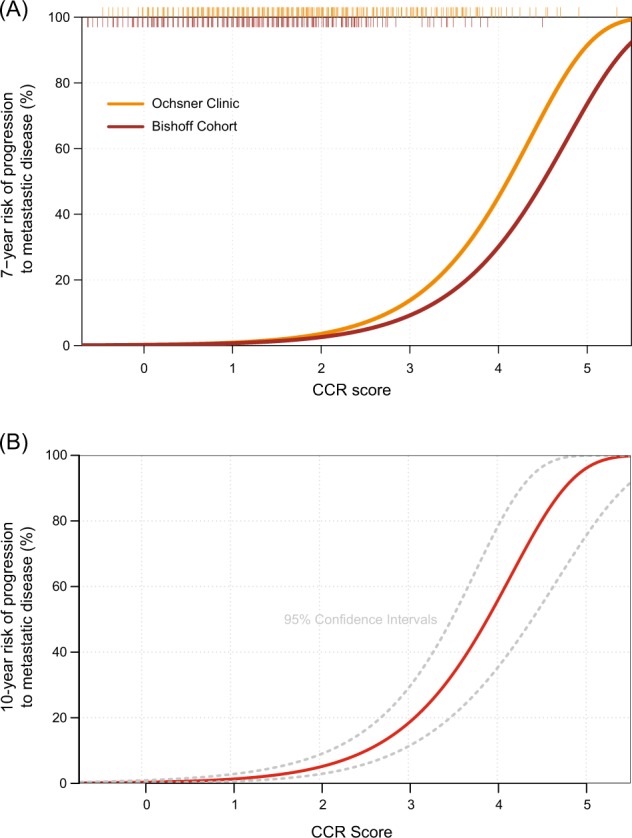
**a** 7-year risk of metastasis according to cohort. **b** 10-year risk of metastasis in the pooled cohort (*N* = 1,062). The rug plot across the top indicates CCR scores for the patients in each cohort

**Fig. 2:**
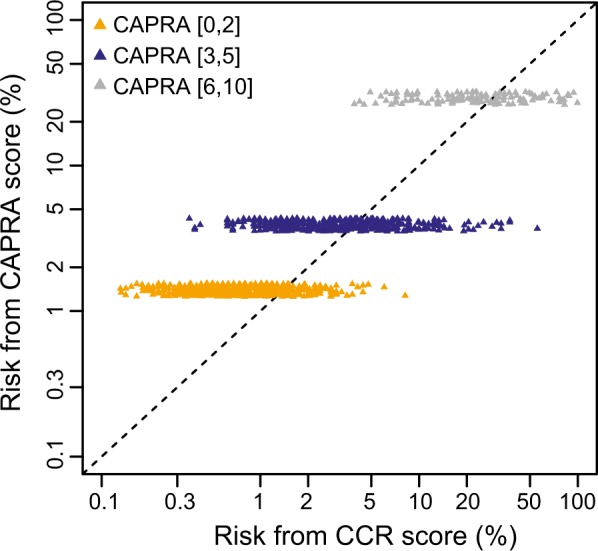
Reclassification of 10-year risk of metastasis using CCR score compared to CAPRA score for men in the pooled cohort (*N* = 1,062). The added prognostic information is illustrated by risk spread along the x-axis

Predicted risk curves showing the 7-year risk of progression to metastatic disease were very similar between cohorts (Fig. 1a), indicating that CCR-based predicted risk is robust regardless of patient composition. The 10-year risk of progression to metastatic disease for the pooled cohort is shown in Fig. 1b. The predicted risks for the cohort ranged from 0.1 to 99.4%, (IQR 0.7%, 4.6%). The amount of added prognostic information provided by CCR is illustrated through comparison of the difference in predicted risk between CCR and a CAPRA-only model (Fig. 2). The additional discrimination is evident by patient spread along the x-axis. The additional prognostic information was also evident when patients were grouped by CAPRA risk category and then stratified by CCP score (Supplemental Fig. 2).

Current prostate cancer guidelines suggest that most low-risk patients should be considered for active surveillance [19, 20]. To ensure that low-risk men did not unduly influence the prediction model, we conducted a sub-analysis using only AUA intermediate and high-risk men. The results were highly comparable. The multivariate HR for progression to metastatic disease for the CCR score was 3.74 (per unit) as compared to 4.00 for the entire cohort. In addition, the predicted risk curves showing the 10-year risk of progression to metastatic disease were nearly identical (Supplemental Fig. 3).

## Discussion

Molecular testing improves risk discrimination in prostate cancer and is recognized in the National Comprehensive Cancer Network guidelines as an important addition to risk stratification for patients with newly-diagnosed prostate cancer [4]. To date, molecular prognostic information derived from biopsy tissue has primarily been used to help inform the decision between immediate treatment or active surveillance [6, 21]. However, prognostic information from the diagnostic biopsy could also be used to help guide treatment intensity in men who should undergo definitive treatment at the time of diagnosis. In this report, we provide evidence from a pooled analysis of several previously published cohorts [9, 12] that the CCP score provides added, independent prognostic information for progression to metastatic disease in men who were treated with either surgery or radiation.

These data demonstrate the prognostic value of the CCR score—a predefined predictive model that combines molecular (CCP score) and clinical (CAPRA score) information. The CCR-based model was highly prognostic and substantially altered predicted risk of metastasis among treated men compared to a CAPRA-only model. The CCR score provided substantial new prognostic information that is not captured by clinical variables included in CAPRA. Importantly, this prognostic information was independent of primary treatment (surgery or radiation ± ADT). As a result, the predicted risks presented here could be used to stratify patients by risk at the time of diagnosis and help direct appropriate treatment planning.

The CCR-based risk curve to predict progression to metastatic disease within 10-years of disease diagnosis could potentially be used to help inform treatment planning. For example, in men considering primary radiation, the predicted risk of progression could aid in determining the extent of the radiation therapy field, or if multimodality therapy is required. Admittedly, there are no data to directly show that treatment intensification will benefit men with high CCR scores, the data presented here indicates that standard interventions were likely to fail for men with high scores. Further study will potentially clarify the clinical utility of increasing intervention intensity in men who appear likely to fail their initial treatment.

The primary limitation of this study is that it was retrospective, which may lead to sample bias. However, all of these cohorts were prospectively collected and sequentially sampled to approximate a disease population-based cohort (with the exception of Intermountain Health Care, which was a case-control cohort). This type of study design and patient sampling should ameliorate most sample bias concerns [22]. Additionally, the samples from the Martini Clinic are not diagnostic biopsies like the other cohorts, but rather simulated biopsies generated from the postoperative block. However, analysis presented in a previous publication [9] and the sensitivity analysis presented here both indicate that these samples did not unduly impact on our conclusions. The retrospective nature of this study may also mean that the predicted event rates are not well calibrated for patients undergoing modern clinical management. Another limitation of the study is that there was no formal way to assess the adherence of the surgeons and radiation oncologists to best-practices that would speak to the quality of the treatments rendered. And finally, this study combined several clinically distinct patient cohorts, which can lead to statistical artifacts. However, we were careful to check for potential cohort effects by adjusting for cohort in all statistical analyses.

The data presented here suggest that molecular prognostic information derived from the diagnostic biopsy could be used to help guide the intensity of primary therapeutic intervention in men with prostate cancer who require definitive treatment. The CCR score was strongly associated with progression to metastatic disease after both surgery and radiation. As such, CCR-based risk stratification may help identify patients who are likely to do well with standard of care, and identify those who may warrant increased intervention intensity due to their predicted risk of metastatic disease.

## Supplementary information

Supplemental Figures
