# Supplementary material for: Analysis of the prognostic utility of the cell cycle progression (CCP) score generated from needle biopsy in men treated with definitive therapy
Source: Prostate Cancer Prostatic Dis. 2019 Jun 27;23(1):102–7. doi: 10.1038/s41391-019-0159-9 (PMC7027968; doi:10.1038/s41391-019-0159-9)

**Supplemental Figure 1.** Forest plot of CCR hazard ratios in a univariate model for the 4 individual cohorts (MC, IHC, DVA, Ochsner) and the combined cohort.


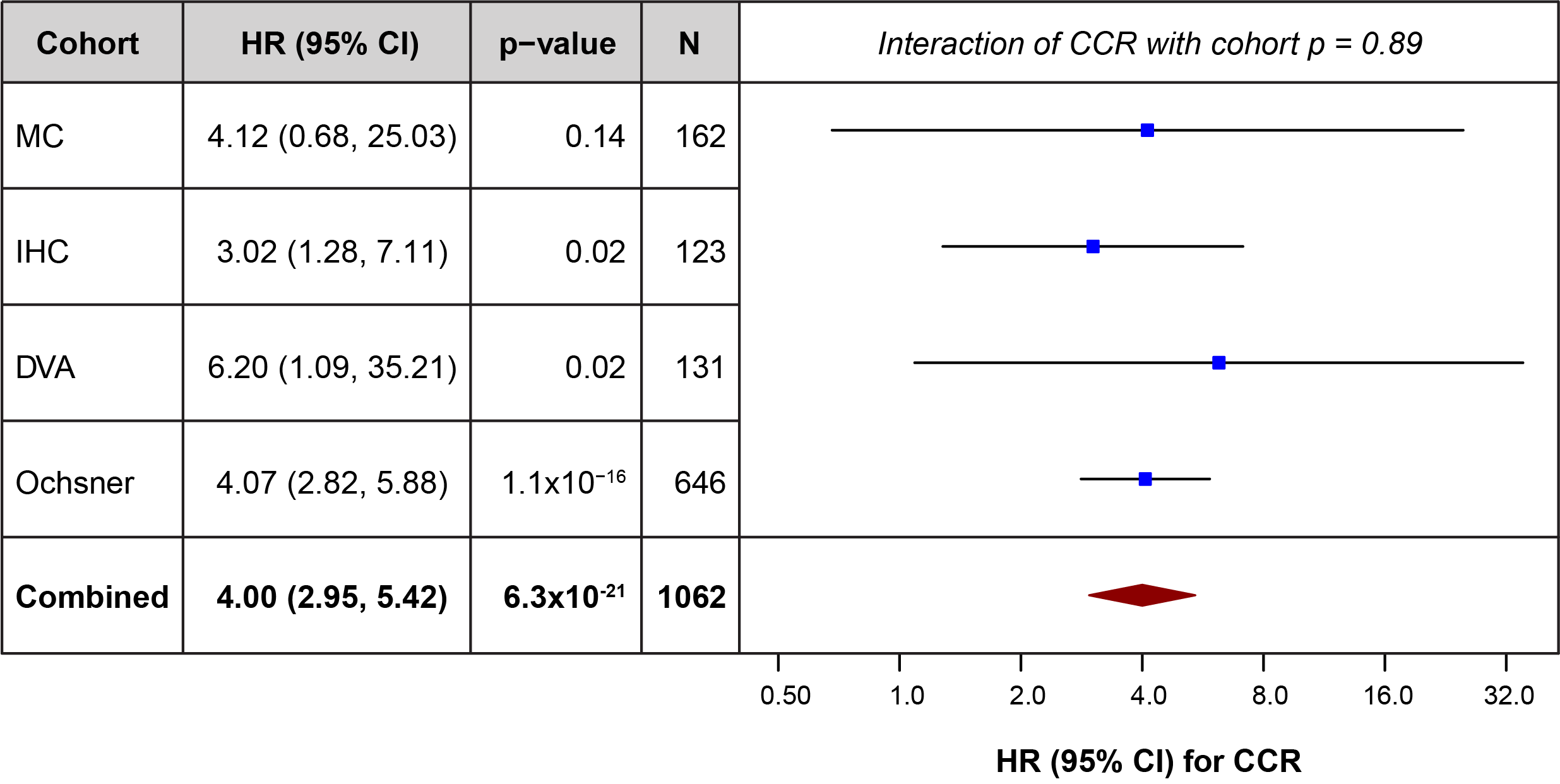


MC: Martini Clinic; IHC: Intermountain Healthcare; DVA: Durham VA Medical Center; HR: hazard ratio; CI: confidence interval.

**Supplemental Figure 2**. Cumulative events stratified by CCP and CAPRA score categories. CCP score provided more information regarding events than CAPRA score alone.


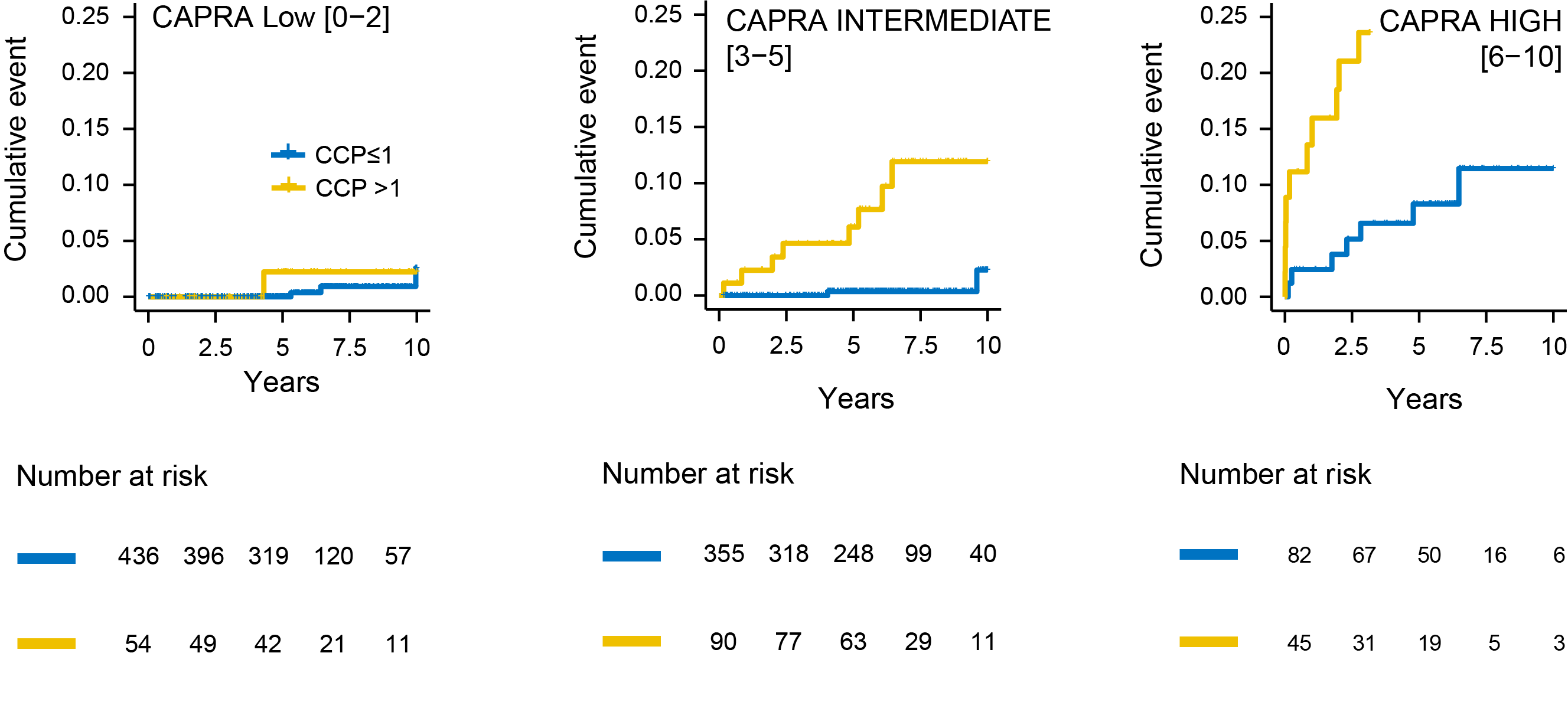


CCP: cell cycle progression score

**Supplemental Figure 3**. 10-year risk of metastatic disease based on the pooled cohort comparing predicted risk derived from all patients to predicted risk using only AUA intermediate- and high- risk patients. The rug plot across the top indicates CCR scores for the patients in each cohort. Note, there was extensive overlap of predicted risks between AUA risk categories


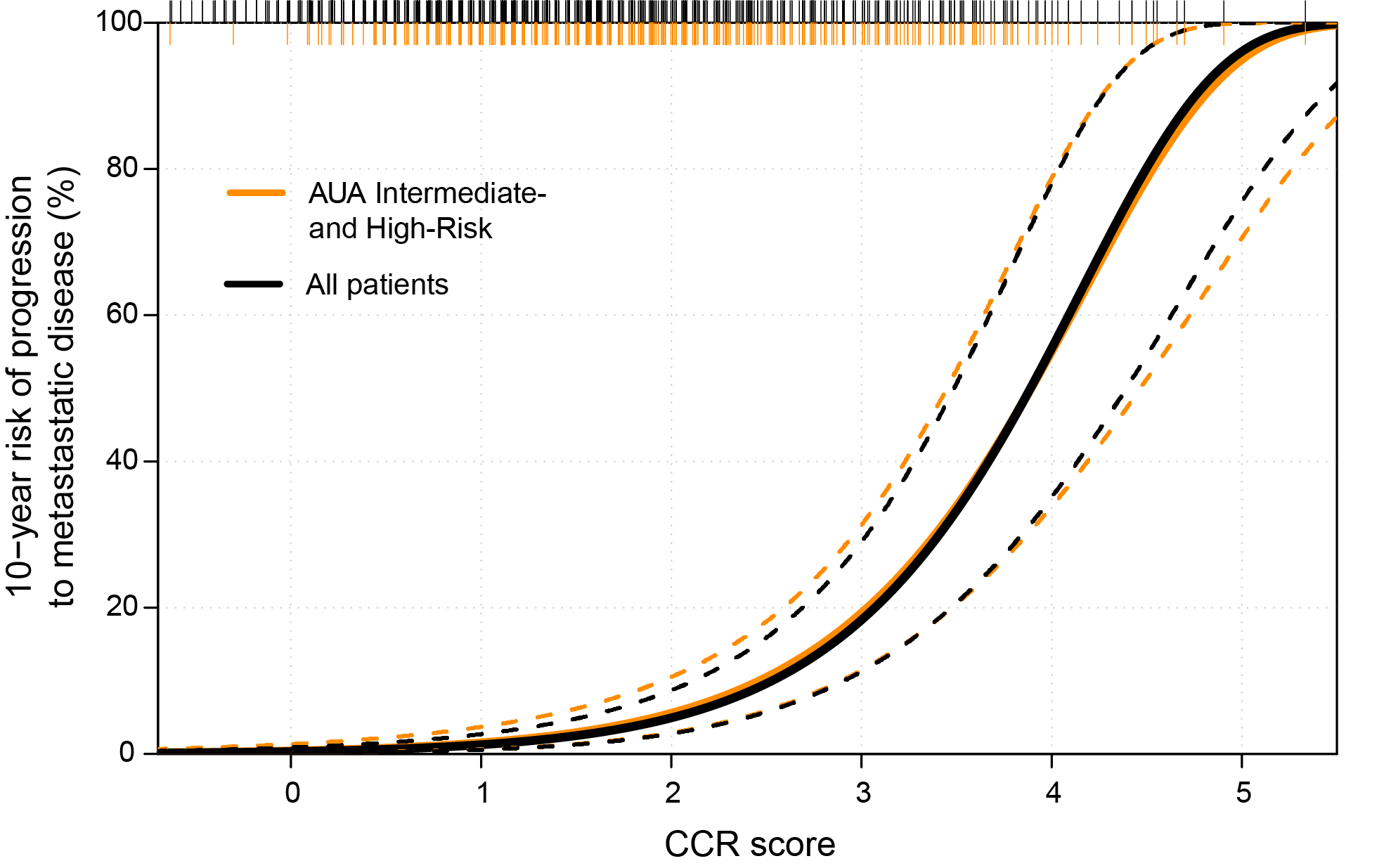

Supplement: Supplementary file 1 — Supplemental Figures [file 41391_2019_159_MOESM1_ESM.docx]
